# Metastatic pancreatic cancer now in remission: a case report and literature review

**DOI:** 10.1007/s12672-025-01756-4

**Published:** 2025-01-09

**Authors:** Charles W. Shi, Thomas J. VanderMeer, Anita Pudusseri

**Affiliations:** 1 SUNY Upstate Medical University, Syracuse, USA; 2 Hematology Oncology Associates of CNY, Syracuse, USA

**Keywords:** Pancreatic adenocarcinoma of the tail, FOLFIRINOX

## Abstract

Pancreatic cancer is a highly aggressive malignancy with the majority of patients presenting at a late stage with unresectable or metastatic disease. Even with first line treatment, median survival is approximately 11 months in patients with advanced PDAC. This report details the unique case of a patient that presented with peritoneal metastases from an adenocarcinoma of the body of the pancreas, had a remarkable response to palliative chemotherapy and is alive without evidence of disease 12 months following cessation of all active treatment. The initial diagnosis was 4 years ago and extensive resection of the primary was completed 2 years ago. The patient was started on standard FOLFIRINOX chemotherapy regimen, completed 6 cycles, but stopped Oxaliplatin and Irinotecan due to neuropathy and fatigue, on November 5, 2020, and transitioned to 5-fluorouracil (5-FU) and leucovorin. There was radiographic response and a notable decrease in tumor marker CA 19-9. On July 12, 2022 he underwent a multivisceral resection that included a radical anterograde modular pancreatico-splenectomy, partial gastrectomy, and splenic flexure colectomy with primary anastomosis due to tumor involvement of the posterior stomach and splenic flexure. Surgical pathology noted a moderately differentiated, grade 2 tumor staged ypT2 N0 M0. He continued the same adjuvant regimen of 5-FU and leucovorin for approximately 9 months with no new or recurrent disease on imaging. His CA 19-9 decreased within normal range after surgery and has remained within the normal limits. He remains on active surveillance. Overall, barring clear availability for targeted therapies, a metastatic PDAC of the tail may be considered to have a better prognosis than previously considered. FOLFIRINOX is the ideal treatment if the patient has a high-performance status, and PRODIGE 35 recommends 8 minimum cycles. However, in our case, the patient only tolerated 6 cycles and was still highly responsive. Despite a stage IV diagnosis, the primary tumor was resected in order to mitigate the risk for mutation and progression. Although rare, greater hope for patients with PDAC of the tail with favorable tumor biology responsive to FOLFIRINOX may contribute to increased surgical resection rates and improve survival rates.

## Introduction

Pancreatic cancer is a highly aggressive malignancy with the majority of patients presenting at a late stage with unresectable or metastatic disease [1, 2]. The estimated 5-year survival rate at the time of diagnosis is 12% in the United States. With a steadily increasing incidence and mortality, it is predicted to become the second leading cause of cancer deaths by 2030 [3, 4]. Most cases are diagnosed as pancreatic ductal adenocarcinoma (PDAC) and locally advanced or metastatic disease is generally considered non-curative and managed with palliative intent [1]. Although significant progress has been made in regards to immune- and targeted systemic therapy and understanding the biology of pancreatic cancer, it has not translated into substantial clinical outcomes for most patients [1, 5, 6]. With regards to first-line chemotherapies, which include FOLFIRONOX (a combination of 5-fluorouracil [5-FU], leucovorin, irinotecan, and oxaliplatin) or gemcitabine plus albumin-bound (nab) paclitaxel, median survival is approximately 11 months [1] in patients with advanced PDAC.

Even in the few patients with an early diagnosis of a localized, resectable tumor, the 5-year survival rate is only about 20% following pancreatoduodenectomy (PD). Additionally, while surgical resection of tumors in the body and tail are less morbid, these tumors are often unresectable due to their late presentation. This report details the unique case of a patient that presented with peritoneal metastases from an adenocarcinoma of the body of the pancreas, had a remarkable response to palliative chemotherapy and is alive without evidence of disease 12 months following cessation of all active treatment. The initial diagnosis was 4 years ago and extensive resection of the primary was completed 2 years ago. The unusual and favorable clinical course of the patient may prompt further investigation to gain insight into treatment and prognosis of this disease.

## Case report

A 59-year-old male with coronary artery disease s/p stents, hypertension, hyperlipidemia, type II diabetes mellitus presented to the emergency department in June 2020 with left flank pain. Computed Tomography (CT) abdomen and pelvis demonstrated diverticulosis and a mass in the tail of the pancreas. Review of systems were significant for intermittent gas, abdominal bloating, and a year-long history of 40 lbs. of unintentional weight loss. He had no jaundice, scleral icterus, lymphadenopathy, or palpable abdominal masses. Family history included a mother who died of unknown cancer at age 48 and a brother diagnosed with colon cancer at age 63. Initial laboratory assessments revealed a normal carcinoembryonic antigen (CEA) at 1.48 ng/mL and an elevated carbohydrate antigen 19-9 (CA 19-9) at 257.46 U/mL. Subsequent CT with pancreatic protocol demonstrated an infiltrative hypo-enhancing pancreatic tail mass measuring 4.3 × 4.2 × 3.9 cm, with encasement of the splenic artery and vein at the level of the mass. There were also scattered nodular peritoneal and omental implants concerning for metastatic disease and an indeterminate left adrenal nodule. CT chest identified multiple calcified and noncalcified granulomatous nodules of the lung bilaterally. Endoscopic ultrasound-guided fine needle aspiration biopsy of the pancreatic mass confirmed ductal adenocarcinoma. He was referred to our practice for initial consultation. Follow-up positive emission tomography-computed tomography (PET-CT) showed physiologic uptake in the chest, a 5.8 × 3.7 cm pancreatic tail mass with SUV of 6.2 and adjacent to the descending colon, a 2.2 × 1.3 cm ill-defined omental nodule with SUV of 3.8. Additional scattered ill-defined omental nodules within the abdomen and pelvis were below the threshold of detection. There was no enhancement of the adrenal or lung nodules. Percutaneous biopsy of the pericolonic nodule confirmed metastatic adenocarcinoma of pancreatic primary and categorized him as stage 4. Of note, comprehensive genetic testing by next-generation sequencing (NGS) had no deleterious alterations or recommended targetable therapies. There were point mutations in ARID1A (Y216*), ATM (S1403fs*11), KRAS (G12D), SMAD4 (F339fs*45), and one variant of uncertain significance in NTHL1 [c.776C > T (p.Pro259Leu)].

The patient was started on standard FOLFIRINOX chemotherapy regimen on August 25, 2020. He completed 6 cycles but stopped Oxaliplatin and Irinotecan due to neuropathy and fatigue, on November 5, 2020, and transitioned to 5-FU and leucovorin. Treatment course was complicated by superior vena cava (SVC) syndrome. There was radiographic response and a notable decrease in tumor marker CA 19-9 (Table 1) until starting a chemotherapy holiday in October, 2021, when CA 19-9 rose from 62.25 to 127.33 U/mL over 4 months. At that time imaging identified no metastatic disease in the liver, peritoneum, small bowel, or regional lymph nodes. CA 19-9 continued to rise, peaking at 197.03  U/mL with no radiographical evidence of disease progression.

**Table 1:** Ca 19-9 levels

Date	Ca 19-9 (U/mL)
7/20/20	257.66
9/4/2020	172.60
10/19/20	148.23
1/12/21	40.36
2/23/21	25.11
7/27/21	42.43
9/7/21	49.73
10/19/21	62.25
2/18/22	127.33
6/8/22	197.03
6/21/22	146.55
7/12/22	Surgery
8/12/22	8.79
9/7/22	8.46
9/27/22	4.63
12/6/22	15.41
1/3/23	11.72
2/10/23	15.63
5/10/23	17.02

With the resolution of the previously noted peritoneal metastases, the patient was re-staged with laparoscopy and peritoneal washings, which were negative. Since the patient seemed to have an unusual response to prolonged chemotherapy, the patient opted for resection. On July 12, 2022 he underwent a multivisceral resection that included a radical anterograde modular pancreatico-splenectomy, partial gastrectomy, and splenic flexure colectomy with primary anastomosis due to tumor involvement of the posterior stomach and splenic flexure. Surgical pathology noted a moderately differentiated, grade 2 2.9 × 2.8x1.8 cm tumor. There was treatment effect present with residual cancer showing evident tumor regression with no LVI, perineural invasion, negative margins, and 0/21 lymph nodes involved (ypT2 N0 M0). He continued the same adjuvant regimen of 5-FU and leucovorin for approximately 9 months with no new or recurrent disease in the abdomen and pelvis on imaging. His CA 19-9 decreased within normal range after surgery and has remained within the normal limits. He remains on active surveillance.

## Methods

Human Ethics Statement: All treatment and procedures performed on this patient were in accordance with institutional standard of care and NCCN guidelines. No experimental protocols were performed. In accordance to SUNY Upstate Medical University Institutional Review Board policy, case reports including less than 3 records, no care was done to the patient with prior research intent, there are no elements of a systematic investigation, the report describes an interesting treatment, presentation, and/or outcome, and the published article will not contain identifiable information with informed consent, and thus receives exemption from IRB approval. All practices were conducted in accordance with the 1964 Helsinki Declaration and all comparable standards.

### Accordance statement

All methods were performed in accordance with the relevant guidelines and regulations as outlined by Springer Nature and Discover Oncology.

Written informed consent was obtained from the patient for publication.

## Discussion

All aspects of this case, including initial staging, location of the primary tumor, and genetic analysis, must be considered in understanding the favorable outcome for this patient. To the authors’ knowledge, only Schneitler et al. have detailed a similar course in 2 patients with PDAC of the tail responding to FOLFIRINOX and having successful R0 resections. However, both of these patients had liver metastases (that resolved) and follow-up CT showed new retroperitoneal LN metastasis within 1 year for one patient^7^. There are also numerous studies and reports on borderline or unresectable locally advanced carcinomas responsive to neoadjuvant chemotherapy, but are limited in the case of definitive metastases and identifying characteristics towards successful tumor regression and resection [7–12]. Metastatic tumor regression has been documented in the presence of BRCA mutations, or in response to immune- and/or targeted therapy, but the clinical application is limited to a small subset of patients and is not applicable to ours [13–16]. NTHL1 is an endonuclease III-like protein 1 that is involved in the base excision repair pathway, repairing oxidative damage in both the nucleus and mitochondria [17]. In a pathogenic mutation, it causes NTHL1 syndrome, a malignancy associated syndrome similar to Lynch syndrome, characterized by an increased risk for colorectal and breast cancer [18]. There is little literature regarding NTHL1 gene expression in pancreatic adenocarcinoma and may represent a further source of investigation in this setting.

For patients with a high-performance status capable of tolerating the toxicity, FOLFIRINOX has been shown increase median overall survival in metastatic disease [1]. The goal is to control the tumor to an operable burden to achieve complete resection. There are no clinical trials supporting surgical resection in metastatic disease, but there are numerous individual reports of attempting resection, most often of the liver, in highly selected patients with varying results [1, 19, 20]. Disease recurrence is 70% following curative resection in metastatic PDAC and has only been found consistently beneficial in the setting of lung metastases [1]. A meta-analysis in 2010 identified resection rates in initially unresectable staging to be 70% after neoadjuvant chemotherapy with a 5-year survival rate comparable to patients capable of immediate resection. However, this included locally advanced and metastatic cancer and was before the introduction of FOLFIRINOX as the standard chemotherapy regimen. Overall, what has continued to stand true is that even in the case of favorable response to neoadjuvant therapy, surgery is riskier due to more extensive resection, as was required in our patient [1, 21]. Notably, Jang et al. identified low contrast enhancement of soft tissue contacting major vasculature to be significantly associated with R0 resection after neoadjuvant FOLFIRINOX [8].

Consideration must also be made towards the location of the primary tumor affecting the prognosis when treated with FOLFIRINOX. A retrospective study by Lorgis et al. describes a primary tumor of the body or tail to be more responsive than that of the pancreatic head, with increased median overall survival of 12 months in metastatic PDAC [22]. However, this difference in response was not identified in previous clinical trial [23]. The latter only included patients with excellent performance status, where 38% of patients had a primary tumor of the pancreatic head, whereas most other trials describe about 70% [22, 23]. This is notable because incidence rates of pancreatic cancer in the body and tail are ever rising [7].

These uncertainties are due to, in large part, a budding understanding of pancreatic cancer biology. Through the known distinct developmental pathways of the pancreatic head, body, and tail, varying malignant potential may arise among the individual cellular environments [7]. In this patient, NGS did not return significant or targetable markers. However, of note, KRAS is mutated in 95% of patients, with the most common being G12D being 42%. There are phase I/II trials in place with KRAS-targeted therapeutics, but little definitive data is present. If recurrence occurs in this patient, as recurrence does occur in 70–80% of patients following surgical resection, use of a KRAS-targeted therapies may be a potential option [1].

Overall, barring clear availability for targeted therapies, a metastatic PDAC of the tail may be considered to have a better prognosis than previously considered. FOLFIRINOX is the ideal treatment if the patient has a high-performance status, and PRODIGE 35 recommends 8 minimum cycles. However, in our case, the patient only tolerated 6 cycles and was still highly responsive, which speaks to the receptiveness of primary tail tumors. Even though the patient had increasing levels of CA 19-9 prior to resection (Table 1.), the primary had shrunk to 2.1 cm on imaging with no radiographic evidence of recurrence. Despite a stage IV diagnosis, the primary tumor was resected in order to mitigate the risk for mutation and progression. Although rare, greater hope for patients with PDAC of the tail with favorable tumor biology responsive to FOLFIRINOX may contribute to increased surgical resection rates and improve survival rates.

## Data Availability

The datasets generated during and/or analyzed during the current study are available from the corresponding author on reasonable request.

## Abbreviations

PDAC: Pancreatic ductal adenocarcinoma
FOLFIRONOX: A combination of 5-fluorouracil, leucovorin, irinotecan, and oxaliplatin
5-FU: 5-Fluorouracil
CT: Computed Tomography
CEA: Carcinoembryonic antigen
CA 19-9: Carbohydrate antigen 19-9
PET-CT: Positive emission tomography-computed tomography
